# High carotenoid production by a halotolerant bacterium, Kocuria sp. strain QWT-12 and anticancer activity of its carotenoid

**DOI:** 10.17179/excli2017-218

**Published:** 2017-06-12

**Authors:** Zahra Rezaeeyan, Atefeh Safarpour, Mohammad Ali Amoozegar, Hamid Babavalian, Hamid Tebyanian, Fatemeh Shakeri

**Affiliations:** 1 Extremophiles Laboratory, Department of Microbiology, Faculty of Biology and Center of Excellence in Phylogeny of Living Organisms, College of Science, University of Tehran, Tehran, Iran; 2 Department of Stem Cells and Developmental Biology, Cell Science Research Center, Royan Institute for Stem Cell Biology and Technology, ACECR, Tehran 19395-4644, Iran; 3 Applied Virology Research Center, Baqiyatallah University of Medical Sciences, Tehran, Iran; 4 Division of Tissue Engineering and Regenerative Medicine, Nanobiotechnology Research Center, Baqiyatallah University of Medical Sciences, Tehran, Iran; 5 Research Center for Prevention of Oral and Dental Disease, Baqiyatallah University of Medical Sciences, Tehran, Iran

**Keywords:** halotolerant bacteria, Kocuria, yellow pigmented bacteria, carotenoid, cancer cell line

## Abstract

Halophilic prokaryotes are extremophile microorganisms that grow optimally in media containing salts and almost appeared pigmented. Many of them contain high concentrations of carotenoids. Amongst 15 strains of halophilic prokaryotes isolated from industrial tannery wastewater in Qom, a Gram-stain-positive coccoid, aerobic, non-endospore-forming, halotolerant bacterium designated as strain QWT-12 showed high capacity in the production of carotenoids in a wide range of culture medium factors. 16S rRNA gene analysis showed that this strain belongs to the genus *Kocuria*. Carotenoids from this strain were extracted by methanol. MTT assay for extracted carotenoid was carried out against seven cancer cell lines belonging to breast, lung and prostate cancer with negative control of fibroblast cell line through six concentration levels to find out IC_50_. Based on statistical analysis of data from MTT assay, IC_50_ of 1, 4 and 8 mg/ml for MCF-7, (A549 and MDA-MB-468) and MDA-MB-231 respectively. Additionally, qualitative carotenoid determination was carried out using spectrophotometric method in 300-600 nm and thin layer chromatography, respectively. According to the obtained results from mass spectrophotometry, absorption spectrum of strain QWT-12 is similar to the absorption spectrum of the carotenoid neurosporene.

## Introduction

Carotenoids are yellow to orange-red pigments widely distributed among nature of many organisms. Their existence happens from bacteria, yeast and algae to animal and plant cells (Basu et al., 2001[3]). Animals are unable to produce carotenoids naturally and presence of carotenoids in their cells is due to their diets which include plant carotenoids (Mortensen, 2006[17]). There are two types of carotenoids structurally, carotenes and xanthophylls. Carotenes group are hydrocarbons without any oxygen atoms while xanthophylls are oxygenated hydrocarbons (de Quirós and Costa, 2006[6]). Almost more than 700 chemical compounds have been classified as carotenoids while about 500 of them have been structurally elucidated. These diverse chemical structures increased functional capability of carotenoids for various biological applications since the exact chemical structure of individual carotenoids is decisive for expected properties and also it determines how they interact with other molecules (Konishi et al., 2008[11]; Irwandi et al., 2011[10]). In addition to antioxidant activity, other applications of carotenoids have been studied as absorbers of light and energy, oxygen transporters, provitamin A, scavengers of active oxygen, antitumor and enhancers of *in vitro* antibody production (Britton et al., 2009[4]). Some of the carotenoids are used commercially as nutritional supplements (lutein), pharmaceuticals (beta-carotene), and food colorants (canthaxanthin) for humans and also as pigments for animal feed (Gordon and Bauernfeind, 1982[9]; Britton et al., 2009[4]). In this competition, new organism producers, innovative processes and well-mixed carotenoid extracts are highly demanded for food nutrition industries (Britton et al., 2009[4]). Additionally, regarding to new reports from medicinal chemists, it has been shown that complex of several discovered carotenoids can have prevention effects against many diseases (Labi et al., 2008[12]) and among them, cancer chemoprevention has been considered the most important part of preclinical investigations that carotenoids showed great functionality to be used in. Diverse carotenoids like β-carotene, lycopene, β-cryptoxanthin, lutein, zeaxanthin, fucoxanthin, canthaxanthin and astaxanthin have been reported to exhibit anticancer effect in both *in vitro* and *in vivo* studies (Tanaka et al., 2012[29]). Many of halotolerant and halophilic microorganisms contain high concentrations of various carotenoids and usually saltern ponds are colored place due to the presence of pigmented microorganisms such as *Dunaliella *which mainly produce β-carotene, haloarchaeal bacterioruberins and some halophilic bacteria such as *Salinibacter *genus that produce salinixanthin carotenoid (Moreno et al., 2012[5]). With the rising global concern to avoid the undesirable effects of synthetic food colorants such as allergy, hypersensitivity, intolerance and childhood hyperactivity (Nguyen, 2013[19]), in this study, we screened halotolerant bacterial strains isolated from tannery wastewater of a company in Qom city in center of Iran, for carotenoid production while effects of different parameters on bacterial growth and carotenoids production of positive ones are also evaluated. MTT assay was done to find the anticancer activity of extracted pigments and then the type of extracted pigments estimated by Thin Layer Chromatography (TLC) and Mass Spectrophotometry (MS). 

## Materials and Methods

### Materials

All the materials and reagents for bacterial cultures and TLC, were purchased from Merck (E. Merck, Darmstadt, Germany).

### Isolation, media and culture collections

Water samples were obtained from tannery wastewater of a company in Qom, Iran. Approximately 1 ml of each sample was poured in 20 ml of nutrient broth medium with 20 % solar salt. The pH was adjusted to 7.2. The flasks were incubated on a shaker at 37 °C until turbidity appeared. The turbid cultures were streaked on nutrient agar 20 % with solar salt and incubated at 37 °C. After 14 day of incubation, yellow colonies were selected and purified (Asker and Ohta, 1999[2]). Amongst 15 isolated halophilic strains, QWT-12, a bacterial strain with sharp yellow colonies were selected for further experiments. Salt tolerance assays for QWT-12 strain were performed by growing it in nutrient broth plus various concentrations of NaCl (0 to 15 % w/v) at 37 °C. The bacterial growth was monitored at temperature range of 10-50 °C with intervals of 5 °C and over a pH values range from 5.0 to 10.0. Sodium acetate buffer was used for pH 6 and lower while Tris-HCl buffer was used for pH higher than 6.

### Identification of the strain

Morphological and physiological characteristics of the strain were either studied on nutrient agar or in nutrient broth plus 3 % (w/v) NaCl. Gram reaction, shape and color of colony, catalase, indole formation and oxidase activities, nitrate reduction and tween 80 hydrolyses were checked as recommended by Smibert and Krieg (1994[27]). Carbon and nitrogen sources utilization were performed according to Ventosa et al. (1982[31]).

Genomic DNA from strain QWT-12 was prepared using the method described by Marmur (1961[14]). The 16S rRNA gene was amplified by PCR with the forward primer 16F27 and the reverse primer 16R1488 as described by Mellado et al. (1995[16]). Direct sequence determination of the amplified DNA was carried out using an automated DNA sequencer (ABI 3130 XL; Applied Biosystems) at the SeqLab Laboratory (Göttingen, Germany). The 16S rRNA molecule phylogenetic analysis was performed using the software package MEGA version 5 (Tamura et al., 2011[28]) after obtaining multiple alignments of data available from public databases (Thompson et al., 1997[30]). Clustering was performed using the neighbour-joining (Saitou and Nei, 1987[25]), maximum-parsimony and minimum-evolution methods. Bootstrap analysis was used to evaluate the tree topology of the neighbour-joining data by performing 1000 resamplings (Felsenstein, 1985[7]).

### Carotenoid production and extraction

Five culture media including TSB, NB, HM, SW and MH mediums were used to find the proper medium for pigment production. The cells were grown at 37 ºC for 24 hours in each medium. After evaluation of optical density and optical pigmentation, TSB medium culture was selected as proper medium to optimize carotenoid production. Additionally, gradient of NaCl, NaNO_3_, Na_2_SO_4_ and KCl in TSB medium were also evaluated to optimize cell growth and pigmentation. Growth curve of strain QWT-12 was drawn in rational to OD and cell pigmentation to find proper time point for pigment extraction.

In order to pigment extraction, the yellow pigmented bacterial isolates were grown in TSB with 3 % (w/v) of salt, in a rotary shaker at 180 rpm in 37 °C for three days. After three days, cells were harvested by centrifugation at 8,000 g for 15 min. Then the pellet was washed with sterile distilled water and spin for 15 min at 4,000 g. Five ml of methanol was added to the pellet and suspended it. Then it was incubated in a water bath at 60 °C for 15 min until all visible pigments were extracted and centrifuged at 4,000 g for 15 min. The colored supernatant was separated, then it was filtered through Whatman no. 1 filter paper. The carotenoid extracts were analyzed by scanning the absorbance in the wavelength region of 450 nm using the spectrophotometer. The total carotenoid content in the methanol extract was estimated by measuring the absorbance at λmax (450 nm) (Ravindar et al., 2014[23]). For MTT assay after evaporating of methanol, the carotenoids were dissolved in ethanol (Abbes et al., 2013[1]).

### Human cell culture

Human cancer cell lines that were used in this project were DU145, PC3, LNCaP for prostate cancer and MDA-MB-468, MDA-MB-231, MCF-7 for breast cancer and A549 for lung cancer. As control cell line we used human skin fibroblast cell line Hu02. All cell lines were purchased from IBRC Cell bank. The cells were grown as a monolayer in an RPMI 1640 medium (Gibco) supplemented with 10 % (v/v) fetal bovine serum (FBS) (Gibco), 1.0 mM sodium pyruvate (Sigma) and 2 mM L-glutamine (Sigma) at 37 °C in a humidified atmosphere of 95 % air and 5 % CO_2_. After 24 h of growth, the cells were transferred into a 96-well plate (2000 cells/well) and treated with the carotenoid extracts for 48 h. The carotenoid extracts were dissolved in RPMI medium and served as a stock solution that was later diluted to have a final solvent concentration.

### MTT assay

The MTT-assay was used to evaluate the carotenoid extract effect on mentioned cell line's cell viability, and the results were expressed as viable cell percentages with respect to the control. Approximately 2000 cells/well were seeded onto a 96-well plate and allowed to adhere for 24 h. Three replicates of each plate were incubated with different concentrations of carotenoid extracts (0.5, 1, 2, 4, 8 mg/ ml), and viability was recorded at 48 h. After treatment, the medium was removed, and 20 μl of 5 mg/ml solution of MTT (Sigma) in PBS were added to each well. The plate was then incubated for 3 h at 37 °C. Finally, the medium was removed, and 200 μl of the DMSO (Merck) were added in each well to solubilize the blue formazan. Dye absorbance was measured at 560 nm (Abbes et al., 2013[1]).

### Carr-price test (Carotenoid test)

The extracted total carotenoids were subjected to TLC using following system: Plate: silica gel 60 F254, Merck; Solvent system: acetone: hexane (40+60); Comparing solutions: regarding to online pigment libraries, stock solution of Neoxanthin, Violaxanthin and Neurosporene were considered to molecular structure alignment (Weber and Davoli, 2003[32]).

A TLC plate, on which the pigment was developed, was sprayed with an absolute chloroform solution of 20 % antimony chloride (III). Mass spectrophotometry test has been carried out in order to align results with online library of similar carotenoids and finally determining the exact structure (Weber and Davoli, 2003[32]).

## Results

### Strain identification

**Figure 1:**
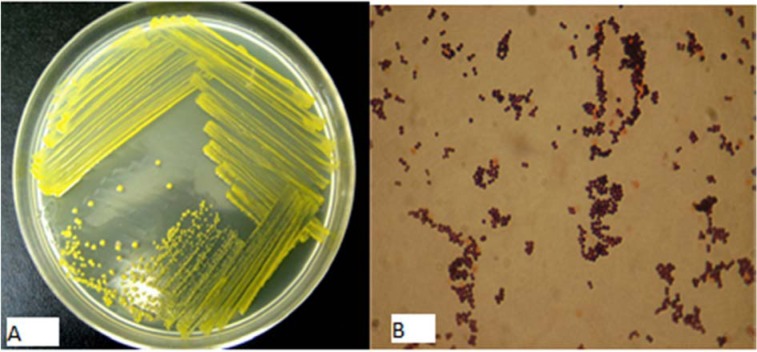
A: QWT-12 colonies under unaided eyes, B: QWT-12 colonies under optical microscope

**Figure 2:**
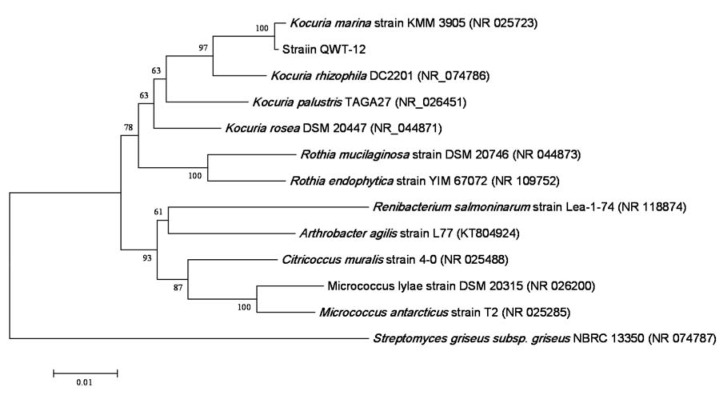
Phylogenetic tree of the QWT-12 strain. Phylogenetic relationships between the QWT12 strain 16S rRNA sequences and other related bacterial sequences. The scale bar corresponds to a 10 % estimated difference in nucleotide sequence positions.

According to phenotypic characterization, strain QWT-12 was a Gram-stain-positive, non-motile, coccus, non-spore forming, facultative aerobic bacterium. Figure 1(Fig. 1) shows the colony of strain QWT-12 on nutrient agar with 3 % salt and optical microscope respectively. The phylogenetic position of strain QWT-12 was determined based on 16S rDNA gene sequencing. 1445 bp of 16S rDNA gene of strain QWT12 with accession no. KX073818 was determined. Alignment of this sequence with described species showed this strain belonged to the genus *Kocuria* with more than 99.8 % similarity to *Kocuria marina* (Figure 2(Fig. 2)). Biochemical characterization results of QWT-12 strain in comparison with *Kocuria marina* KMM 3905^T ^have been exhibited in Table 1(Tab. 1).

### Effect of various factors on pigment production

**Figure 3:**
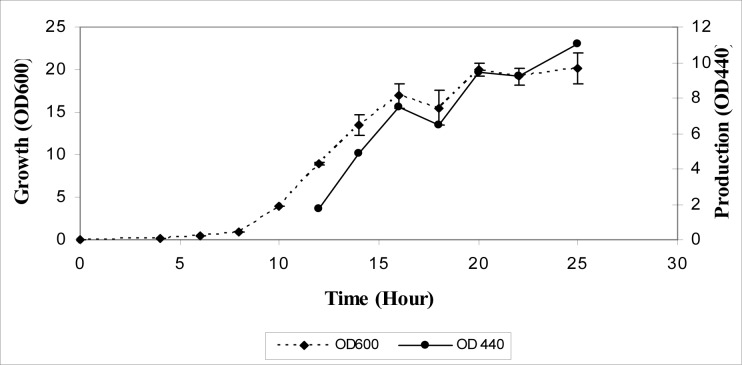
Growth curve for QWT-12 in relation with OD and cell pigmentation

Kinetics of bacterial growth and pigment production were investigated in the nutrient broth plus 3 % (w/v) NaCl. Lag phase of the bacterial growth was 15 h and after 23 h, growth reached stationary phase. Pigment production activity was undetectable during the early- and mid-exponential growth phase but started from the end of the exponential phase and continued in the stationary phase (Figure 3(Fig. 3)). To determine the optimal growth conditions yielding the highest pigment production activity, the effect of various culture media was analyzed.

**Figure 4:**
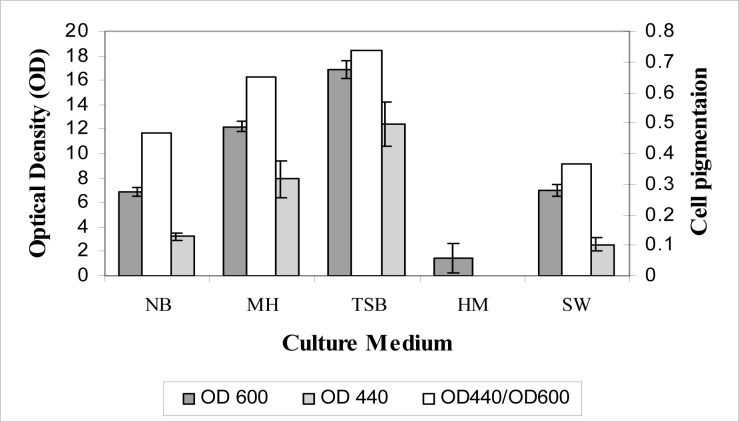
Effects of NB, MH, TSB, HM and SW culture mediums on cell pigmentation and OD

**Figure 5:**
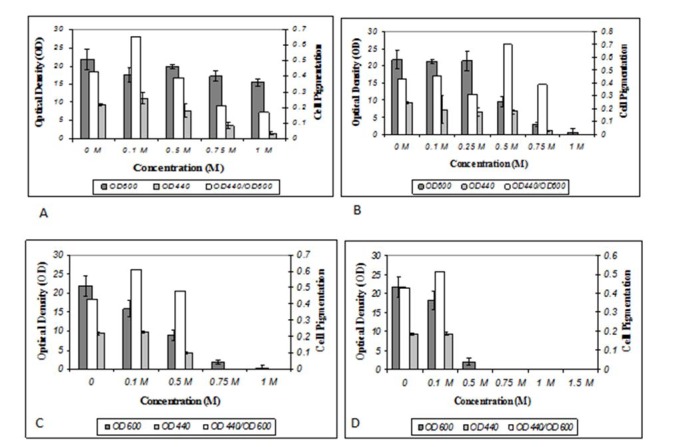
A: KCl gradient effect, B: NaCl gradient effect, C: NaNO_3_ gradient effect, D: Na_2_SO_4_ gradient effect

In Figure 4(Fig. 4), comparative effectiveness of each culture medium for cell growth and cell pigmentation were exhibited elaborately. Of the five culture media investigated TSB medium was the best for growth and pigment production. Effects caused by gradient of NaCl, NaNO_3_, Na_2_SO_4 _and KCl in TSB medium on cell pigmentation and OD were shown in Figure 5(Fig. 5). According to these results the proper concentration of different salts for pigment production and growth was 0.1 M for NaNO_3_, Na_2_SO_4 _and KCl and 0. 5 M for NaCl.

### MTT assay and IC_50_ analysis

**Figure 6:**
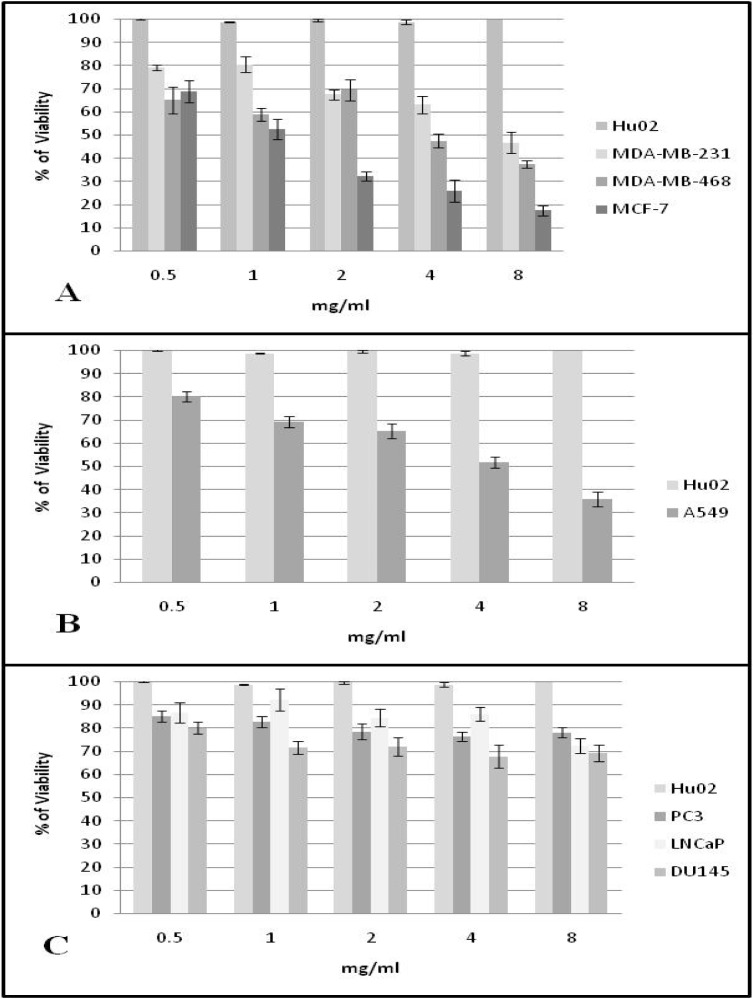
MTT assay results for five gradient levels of *Kocuria* strain QWT-12 carotenoid against fibroblast cell line (Hu02) and A: Breast cancer cell lines, B: Lung cancer cell line and C: Prostate cancer cell lines

In Figure 6(Fig. 6) viability percentage results gained from using five gradient concentration levels of *Kocuria* carotenoid against seven cancer cell lines have been shown. Abbreviations located beside histogram are due to cell lines while Hu02 has been considered negative control. After calculation for the chi-square test significant IC_50s_ of 1, 4 and 8 mg ml of extracted pigments against MCF-7, (A-549 and MDA-MB-468) and MDA-MB-231 cancer cell lines were obtained respectively. The extracted pigments have no effect on viability of fibroblast cell line and only 20- 30 percent decrease the viability of prostate cancer cell lines.

### Pigment analysis

**Figure 7:**
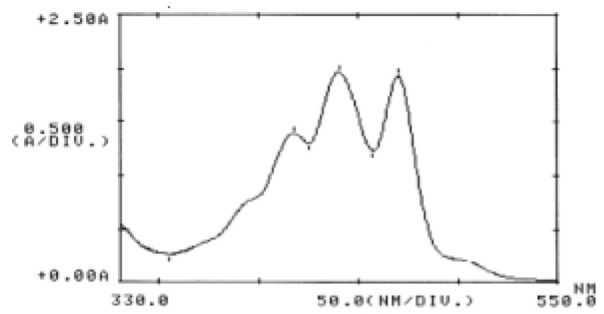
UV/Visible spectra of yellow pigments in n-hexane solvent

Extracted pigments were separated into several spots by TLC. The orange colored pigment which had the highest affinity for the mobile (solvent) phase migrated towards the top of the TLC, and had Rf equal to the obtained Rf from stock solutions. Two other extracted pigments were also present, including the yellow pigments which have the higher affinity for the stationary (silica) phase. The carr-price test for all spots confirming that these pigments are carotenoids. The UV/light absorption spectra of two yellow pigments were identical to those violaxanthin and neurosporene (Figure 7(Fig. 7)). The absorption maxima of extracted pigments in different solvents including chloroform, ethanol and petroleum ether in comparison with neoxanthin, violaxanthin and neurosporene were exhibited in Table 2(Tab. 2). Number of conjugated double bonds (N) can be estimated following the formulae obtain from (Rivera et al., 2014[24]).

In ethanol:


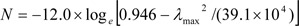


For both two pigments, it was found that N=9 and according to chemical structure of neoxanthin (N=8), violaxanthin (N=9) and neurosporene(N=9) we could estimate that these extracted pigments are violaxanthin or neurosporene.

### Mass spectrophotometry

**Figure 8:**
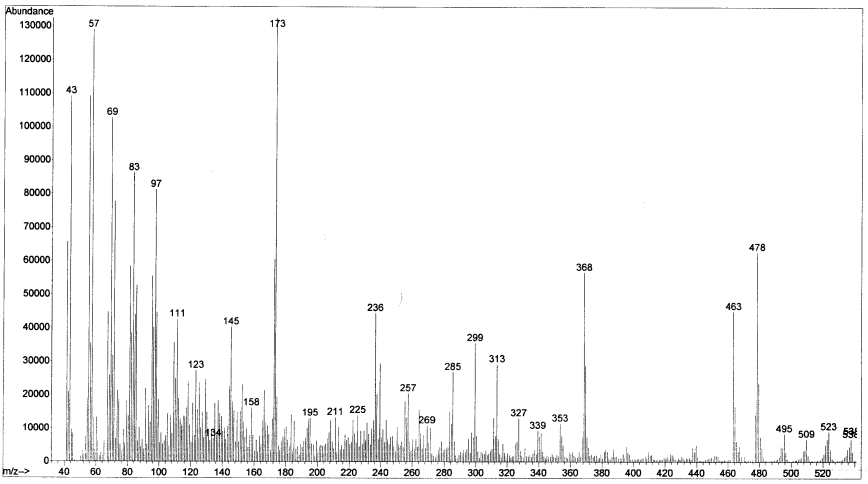
Mass spectrophotometry of yellow pigment extracted from TLC

To more structure elucidation of the extracted pigments, mass spectrophotometry was done for the yellow pigment, as it seems to be the major part of the pigment extraction. Figure 8(Fig. 8), exhibited that the molecular weight (MW) of yellow pigment (yielded from TLC plate) is 538 and this has more similarity to neurosporene (MW=538.8) than violaxanthin and neoxanthin (MW=600). According to all above, it could be estimated that the principle pigment extracted form *Kocuria* sp. strain QWT-12 is neurosporene. 

## Discussion

Halophiles which inhabit almost in hypersaline environments like saline ponds, showed great potential toward production of various carotenoids including *Dunaliella, *producer of beta-carotene, *Salinibacter ruber*, rich in salinixanthin (de Lourdes Moreno et al., 2012[5]). There exist many factors which influence carotenoid production by microbial cell factory and the most important and highly affecting ones especially in halophiles is salinity of culture condition since, adapting to salinity requires and also leads to altering in metabolic pathways. Hence the possibility of carotenoid over-production could be handled resourcefully (Margesin and Schinner, 2001[13]). Naziri et al. (2014[18]) exhibited optimal total carotenoid production by *Halorubrum* sp. TBZ126 had occurred in 16.55 % and 20.55 % sodium chloride concentration by using Response Surface Methodology. In this present study, not only we have shown that the maximum carotenoid extraction has correlated to 3 % salinity but also we opted that the most appropriate culture media for cell factory optimization is TSB medium. 

Among different carotenoids, neurosporene has commercial importance due to its use as an antioxidant and UV-B radiation protector. It is a tetra-terpenoid carotenoid commonly found in plants and vegetables and also in bacteria, fungi, algae and serves as a precursor for more than 600 carotenoids (Sandmann et al., 1999[26]). This carotenoid is not commercially available at present and hence greatly depends on dietary supplements (Nollet and Toldrá, 2012[20]). We found that the halotolerant *Kocuria* sp. strain QWT-12 has pigment likewise neurosporene pigments as major carotenoid pigment. Although neurosporene is an intermediate in carotenoid synthesis, its accumulation exhibited in a mutant strain of *Rhodobacter capsulatus *(Giuliano et al., 1988[8]; Ramaprasad et al., 2013[22]). 

Recent preclinical and medicinal studies have shown inverse relation between dietary uptake of carotenoids and risk factors belonged to cancer and tumor occurrence. Not only *in vivo* studies confirm chemoprevention effects of some carotenoids but also *in vitro* studies support it by many tissue modeling anticancer activity (Tanaka et al., 2012[29]). Based on current research we exhibited that MTT assay for extracted *Kocuria* sp. strain QWT-12 carotenoid possess the IC_50_ against MCF-7, A549, MDA-MB-468 and MDA-MB-231 in 2, 4 and 8 mg/ml concentrations. The cancer preventive potential of carotenoids has been reported in several studies involving cultured cells and animal models (Martini et al., 2010[15]). Carotenoids have been shown to suppress the cancer cell *in vitro,* by inducing differentiation and apoptosis and cell cycle arrest. Several studies exhibited the viability decrease of several cancer cell lines including colon, melanoma, prostate, oral, lung, and breast cancer cells in presence of different type of carotenoids (Palozza et al., 2003[21]). Abbes et al. (2013[1]) also demonstrated that carotenoid extract of a halophile Archaea, *Halobacterium halobium *showed significant antiproliferative activity against HepG2 human cancer cell lines.

## Conclusion

Although fermentation of carotenoids through microbial cell factory has not reached yet to yield due to plant natural carotenoids but a great spectrum of microbial carotenoids is identified and extracted increasingly and our research established cutting edge ideas toward both preclinical studies and screening for anticancer activity of natural products.

## Figures and Tables

**Table 1:**
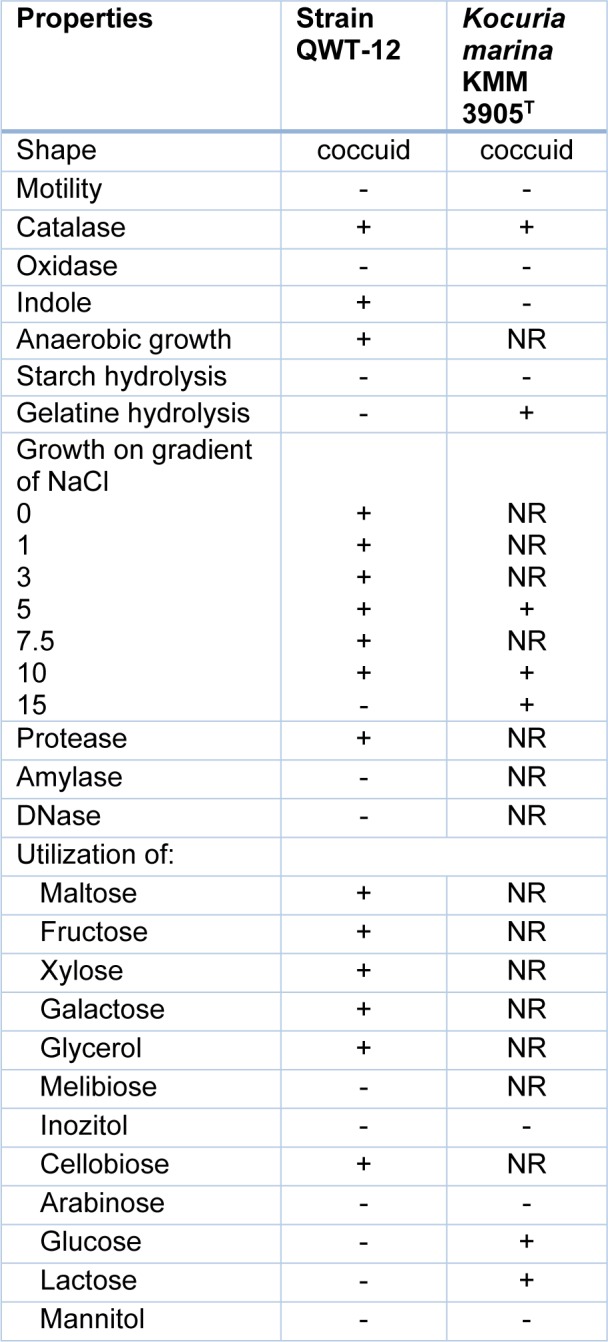
Biochemical test results due to QWT-12 characterization. +; Positive, -; Negative

**Table 2:**
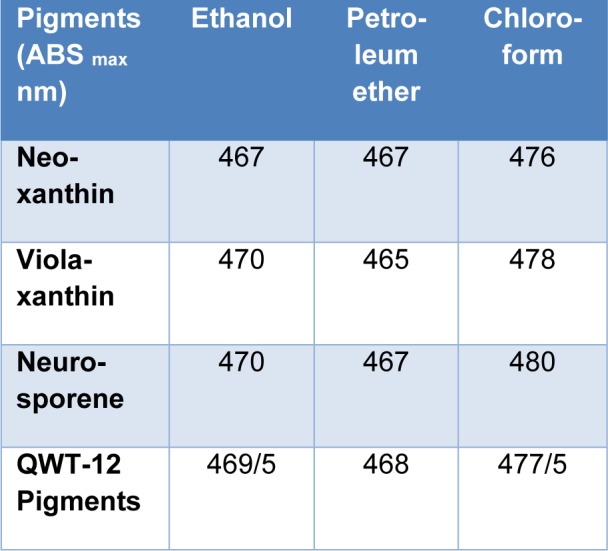
Light absorption maxima of QWT-12 pigments in comparison with Neoxanthin, Violaxanthin and Neurosporene
