# High carotenoid production by a halotolerant bacterium, Kocuria sp. strain QWT-12 and anticancer activity of its carotenoid

**DOI:** 10.17179/excli2017-218_corrigendum

**Published:** 2017-11-07

**Authors:** Zahra Rezaeeyan, Atefeh Safarpour, Mohammad Ali Amoozegar, Hamid Babavalian, Hamid Tebyanian, Fatemeh Shakeri

**Affiliations:** 1 Extremophiles Laboratory, Department of Microbiology, Faculty of Biology and Center of Excellence in Phylogeny of Living Organisms, College of Science, University of Tehran, Tehran, Iran; 2 Department of Stem Cells and Developmental Biology, Cell Science Research Center, Royan Institute for Stem Cell Biology and Technology, ACECR, Tehran 19395-4644, Iran; 3 Applied Virology Research Center, Baqiyatallah University of Medical Sciences, Tehran, Iran; 4 Division of Tissue Engineering and Regenerative Medicine, Nanobiotechnology Research Center, Baqiyatallah University of Medical Sciences, Tehran, Iran; 5 Research Center for Prevention of Oral and Dental Disease, Baqiyatallah University of Medical Sciences, Tehran, Iran

## Corrigendum

Corrigendum to manuscript

**HIGH CAROTENOID PRODUCTION BY A HALOTOLERANT BACTERIUM, KOCURIA SP. STRAIN QWT-12 AND ANTICANCER ACTIVITY OF ITS CAROTENOID**

EXCL Journal 2017;16:840-851

840

Unfortunately, the list of authors of the aforementioned article was incorrect. The correct list of authors is:

Zahra Rezaeeyan^1^, Atefeh Safarpour^2^, Mohammad Ali Amoozegar^1,*^, Hamid Babavalian^3^, Hamid Tebyanian^4, 5^, Fatemeh Shakeri^4^

1 Extremophiles Laboratory, Department of Microbiology, Faculty of Biology and Center of Excellence in Phylogeny of Living Organisms, College of Science, University of Tehran, Tehran, Iran

2 Department of Stem Cells and Developmental Biology, Cell Science Research Center, Royan Institute for Stem Cell Biology and Technology, ACECR, Tehran 19395-4644, Iran

3 Applied Virology Research Center, Baqiyatallah University of Medical Sciences, Tehran, Iran

4 Division of Tissue Engineering and Regenerative Medicine, Nanobiotechnology Research Center, Baqiyatallah University of Medical Sciences, Tehran, Iran

5 Research Center for Prevention of Oral and Dental Disease, Baqiyatallah University of Medical Sciences, Tehran, Iran

* corresponding author: Mohammad Ali Amoozegar, Extremophiles Laboratory, 

Department of Microbiology, Faculty of Biology and Center of Excellence in Phylogeny 

of Living Organisms, College of Science, University of Tehran, Tehran, Iran, 

Telephone: +98 21 61113557, Fax: +98 21 23562507, E-mail: amoozegar@ut.ac.ir 

http://dx.doi.org/10.17179/excli2017-218

http://dx.doi.org/10.17179/excli2017-218_corrigendum

This is an Open Access article distributed under the terms of the Creative Commons Attribution License 

http://creativecommons.org/licenses/by/4.0/.

